# Effect of variable breath-hold positions during cardiac magnetic resonance on measures of left ventricular mechanics

**DOI:** 10.1186/1532-429X-16-S1-P78

**Published:** 2014-01-16

**Authors:** Sean M Hamlet, Gregory J Wehner, Jonathan D Suever, David Powell, Christopher M Haggerty, Linyuan Jing, Xiaodong Zhong, Frederick H Epstein, Brandon K Fornwalt

**Affiliations:** 1 Pediatrics, University of Kentucky, Lexington, Kentucky, USA; 2 Biomedical Engineering, University of Kentucky, Lexington, Kentucky, USA; 3 Electrical Engineering, University of Kentucky, Lexington, Kentucky, USA; 4 Biomedical Engineering, University of Virginia, Charlottesville, Virginia, USA; 5 MR R&D Colloborations, Siemens Healthcare, Atlanta, Georgia, USA

## Background

Measures of left ventricular cardiac mechanics such as strains and torsion are becoming increasingly important for assessing heart function. Cardiac magnetic resonance (CMR) can be used to quantify cardiac mechanics using several methods such as tagged CMR or cine Displacement Encoding with Stimulated Echoes (DENSE). These images are generally acquired during an end-expiratory breath-hold. Unfortunately, it is difficult for subjects to hold their breath at the exact same position when undergoing a series of breath-holds during a typical CMR study. For example, end-expiratory breath-hold positions have an average range of about 8 millimeters (mm). The effects of different breath-hold positions on measures of cardiac mechanics have not been investigated. We hypothesized that the normal variability in breath-hold positions would significantly affect the quantification of left ventricular strains and torsion.

## Methods

Eight healthy volunteers (age 27 ± 3 years) with no history of cardiovascular disease were consented. A 3T Siemens Tim Trio scanner was equipped with a navigator feedback system to enable subjects to view their diaphragm position in real time during image acquisition. We used this navigator feedback system to acquire a navigator-gated basal, mid-ventricular, and apical slice of two-dimensional cine DENSE at three different breath-hold positions spaced 4 mm apart for a total range of 8 mm. The 2D DENSE parameters were: 6 spiral interleaves, FOV = 340, matrix = 128 × 128, thickness = 8 mm, TE/TR = 1.08/17, flip angle = 20, averages = 1. A narrow navigator acceptance window of ± 2 mm was used to properly simulate the different diaphragm positions. Radial strains, circumferential strains, and torsion were calculated for each subject and compared between diaphragm locations using a repeated measures ANOVA with a Huynh Feldt correction.

## Results

**Figure 1:**
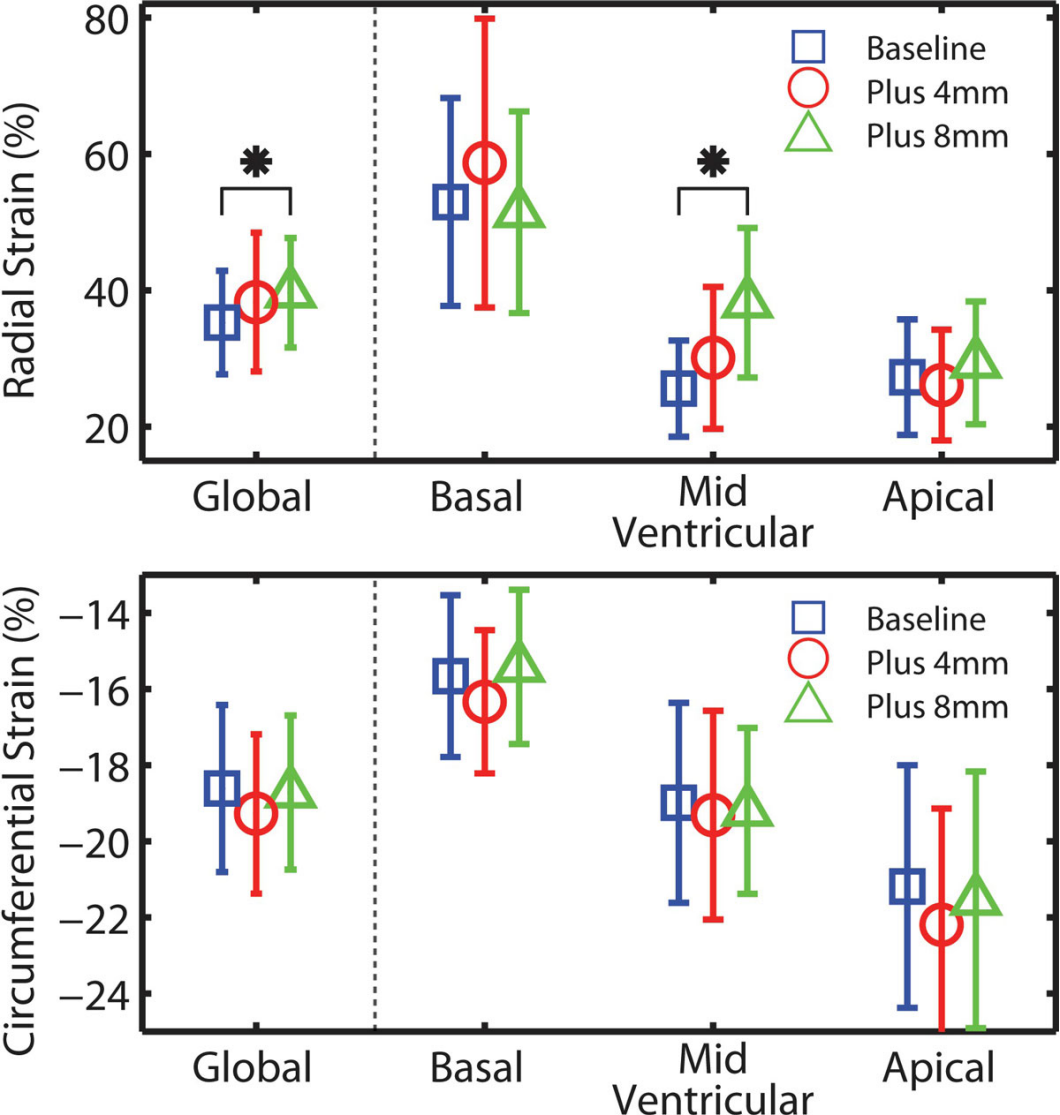
**Average (global) radial and circumferential peak left ventricular strain at the basal, mid-ventricular and apical locations in the heart for 3 different breath-hold positions: baseline, plus 4 mm, and plus 8 mm**. Bars indicate the standard deviations. * indicates p < 0.05 when comparing the three breath-hold positions.

Diaphragm position had a minimal effect on left ventricular radial strain, circumferential strain, and torsion (Figure 1). The only significant difference between navigator positions was for the mid-ventricular and global peak radial strains (p = 0.01 for both). Estimated power for detecting a difference was adequate between 70 and 100% suggesting a low probability for Type II errors.

## Conclusions

Different breath-hold positions simulated with a navigator feedback system had minimal effects on the calculation of peak left ventricular cardiac strains and torsion from two-dimensional DENSE CMR. It will be important to determine whether this result holds true in a future study which includes patients with potentially heterogeneous contraction patterns in the left ventricle.

## Funding

NIH Early Independence Award to BKF (DP5 OD012132) University of Kentucky Cardiovascular Research Center, grant UL1RR033173 from the National Center for Research Resources (NCRR), funded by the Office of the Director, National Institutes of Health (NIH) and supported by the NIH Roadmap for Medical Research Contributions made by local businesses and individuals through a partnership between Kentucky Children's Hospital and Children's Miracle network. The content is solely the responsibility of the authors and does not necessarily represent the official views of the funding sources.

